# Cone snail species off the Brazilian coast and their venoms: a review and update

**DOI:** 10.1590/1678-9199-JVATITD-2022-0052

**Published:** 2023-01-27

**Authors:** Helena B. Fiorotti, Suely G. Figueiredo, Fabiana V. Campos, Daniel C. Pimenta

**Affiliations:** 1 Laboratory of Biochemistry and Biophysics, Butantan Institute, São Paulo, SP, Brazil.; 2 Graduate Program in Biochemistry, Laboratory of Protein Chemistry (LQP), Federal University of Espírito Santo, Vitória, ES, Brazil.

**Keywords:** Conus, Cone snail, Brazilian coast, Venom, Conopeptides, Conotoxins

## Abstract

The genus *Conus* includes over 900 species of marine invertebrates known as cone snails, whose venoms are among the most powerful described so far. This potency is mainly due to the concerted action of hundreds of small bioactive peptides named conopeptides, which target different ion channels and membrane receptors and thus interfere with crucial physiological processes. By swiftly harpooning and injecting their prey and predators with such deadly cocktails, the slow-moving cone snails guarantee their survival in the harsh, competitive marine environment. Each cone snail species produces a unique venom, as the mature sequences of conopeptides from the venoms of different species share very little identity. This biochemical diversity, added to the numerous species and conopeptides contained in their venoms, results in an immense biotechnological and therapeutic potential, still largely unexplored. That is especially true regarding the bioprospection of the venoms of cone snail species found off the Brazilian coast - a region widely known for its biodiversity. Of the 31 species described in this region so far, only four - *Conus cancellatus*, *Conus regius*, *Conus villepinii*, and *Conus ermineus* - have had their venoms partially characterized, and, although many bioactive molecules have been identified, only a few have been actually isolated and studied. In addition to providing an overview on all the cone snail species found off the Brazilian coast to date, this review compiles the information on the structural and pharmacological features of conopeptides and other molecules identified in the venoms of the four aforementioned species, paving the way for future studies.

## Background

The genus *Conus* Linnaeus, 1758, a member of the Conidae J. Fleming, 1822 family, comprises a group of marine venomous snails, with more than 900 species recognized to date [1]. The so-called cone snails are distributed throughout the globe, predominantly in tropical waters. These animals usually dwell near reefs, burying in the sand during the day and hunting at night. 

Cone snails are usually divided into three groups according to feeding habit: the vermivorous group, which feeds on worms; the molluscivorous group, which hunts other gastropods; and the piscivorous group, the fish-hunting cone snails. Among the immense diversity of cone snail species, those belonging to the piscivorous group pose a greater threat to humans, being responsible for serious and sometimes fatal accidents [2]. Nevertheless, molluscivorous and vermivorous cone snails also produce toxins that can be harmful to vertebrate systems, at the very least under experimental conditions [3, 4].

Through millions of years of evolution, cone snails developed a highly sophisticated and well-conserved venom apparatus for prey capture and defense against predators [5, 6]. It consists of a duct, where the venom is synthesized and stored, a bulb, which transfers venom from the duct [7], and a hollow, harpoonlike radula tooth, which enables the fast and efficient delivery of venom into the prey [8].

**Figure 1.**
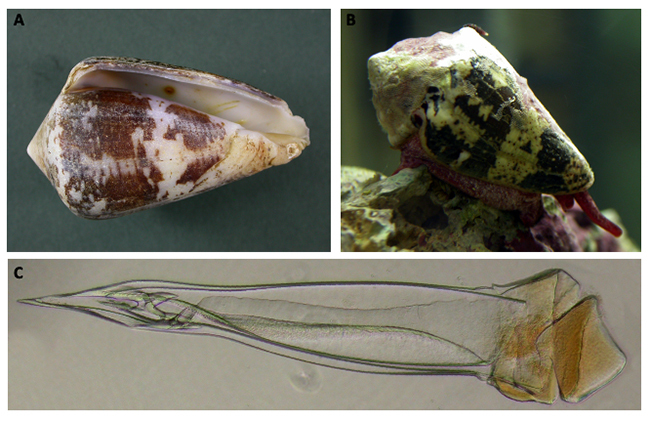
Examples of cone snail species found off the Brazilian coast. **(A)** *Conus ermineus* shell*.* MNRJ 8741. 70.2 x 40.4 mm. **(B)** *Conus regius* live specimen. Rio de Janeiro National Museum collection (MNRJ) 9704. 47 mm. Photos by Paulo Márcio Costa. **(C)** *Conus regius* radula tooth. MNRJ 9608. Optical microscopy photo (200x) by Renata Gomes.

A shell of the piscivorous species *Conus ermineus* and a live specimen of the vermivorous *Conus regius* and its radula tooth are shown in Figures 1A, 1B, and 1C, respectively. Both species, although not endemic, are found off the Brazilian coast and the exploration of their venoms will be discussed in this review. 

The composition of the venom produced by cone snails depends on the purpose it must serve, that is, defense or predation, and the protein expression pattern along the venom duct is closely related to the type of venom produced [9]. Regardless, cone snail venoms are highly efficient weapons because their major components - small toxic peptides with about 10 to 45 amino acid residues named conopeptides - have ion channels and membrane receptors as canonical pharmacological targets [10, 11]. There have been also some reports about the presence of other bioactive compounds in cone snail venoms with hormone-like [12-14], proteolytic [15-17], hyaluronidase [18, 19] and phospholipase [20] activities, in addition to small non-peptidic molecules such as neurotransmitters [21, 22]. However, these cone snail venom components are less studied than conopeptides. 

Conopeptides act with a high degree of specificity, selectively disturbing crucial physiological processes that involve electrical signaling and signal transduction as a whole. For instance, the venom of fish-hunting species that use the hook-and-line strategy contains conopeptides that act synergistically to quickly immobilize and paralyze the prey - the “lightening-strike” and motor cabals, respectively [23]. This cocktail simultaneously inhibits the inactivation of neuronal Na^+^ channels and blocks skeletal muscle Na^+^ channels, K^+^ channels, presynaptic Ca^2+^channels, and nicotinic acetylcholine receptors (nAChRs) [24]. Those who employ the “net-engulfment” strategy first release a “nirvana” cabal into the water, which contains an insulin-like peptide and other components that numb the fish, to which follows the injection of the paralytic motor cabal [13]. Thus, by producing a biochemically engineered venom that acts in different systems, cone snails guarantee their survival in the diverse and very competitive marine environment.

Traditionally, conopeptides are divided into two broad groups: disulfide-poor peptides, a minor group whose members have a single disulfide bond or even none at all; and disulfide-rich peptides that contain two or more disulfide bonds, also known as conotoxins [10]. The first group includes conopeptides with canonical targets, hormone-like conopeptides, and conopeptides whose targets remain unknown [25]. A list of disulfide-poor conopeptide families, along with their known/potential targets, is displayed in Table 1.

**Table 1.** Disulfide-poor conopeptides and their known targets.

Family	General target
Conantokins^0^	NMDA receptor
Conorfamides^0^	RFamide receptor, ASIC channels, nAChR
Conolysins^0^	Cellular membranes
Conophans^0^	ND
Conomaps^0^	ND
Conomarphins^0^	ND
Cono-NPYs^0^	ND
Contulakins^0^	Neurotensin receptor
Hormone-like conopeptides^0^ (Elevenin, PH4)	ND
Contryphans^1^	K^+^ and Ca^2+^ channels
ConoGAYs^1^	ND
ConoCAPs^1^	ND
Conopressins^1^	Vasopressin/oxytocin receptor

^0^ No disulfide bond; ^1^one disulfide bond; ND: not determined.

The conotoxins group is much more complex, as each cone snail species produces a unique venom containing hundreds of different such peptides. This uniqueness arises mainly from the fact that, except for the number and position of cysteine residues, which are conserved among conotoxins that share the same cysteine framework and disulfide connectivity, the mature sequences of conotoxins from different species share very little identity [10]. Nevertheless, the gene superfamilies - defined by conserved signal sequences - that encode such a diversity of conotoxins are relatively few, and a same cysteine framework can be shared by different superfamilies [26]. Conotoxins differ also in the length of the loops formed by the residues that flank the conserved cysteine residues, in a classification system known as loop class [27]. They can be also classified according to three-dimensional structure, with different folds (A-L and Kunitz fold) and sub-folds being determined mainly by disulfide connectivity [27]. However, conotoxins that belong to different gene superfamilies and display different disulfide patterns, loop classes, and 3D structures can have the same target, which would classify them into the same pharmacological family [26, 27]. As an overlap between these classification schemes does not always take place, the task to group the ever-growing number of conotoxins described into simple categories is an impossible one. 

The gene superfamilies, cysteine frameworks, pharmacological families, and targets of conotoxins described so far are listed in Table 2. For simplicity’s sake, we chose to overlook the other classification systems in this list. We must highlight that Table 2 does not include conodipines, which display phospholipase (PLA_2_) activity, and con-ikot-ikot, which target a-amino-3-hydroxy-5-methyl-4-isoxazolepropionate (AMPA)-type glutamate receptors, both dimeric cysteine-rich conotoxins that do not fall into known gene superfamilies [20, 28].

**Table 2.** Gene superfamily, cysteine framework, general targets, and pharmacological families of conotoxins according to ConoServer [26].

Superfamily	Cysteine framework	Pharmacological family	General Target
A	I (CC-C-C)	α	Nicotinic acetylcholine receptors (nAChR)
		ρ	Alpha1-adrenoceptors (GPCR)
	II (CCC-C-C-C)	α	Nicotinic acetylcholine receptors (nAChR)
	IV (CC-C-C-C-C)	α	Nicotinic acetylcholine receptors (nAChR)
		κ	K^+^ channels
		μ	Na^+^ channels
	VI/VII (C-C-CC-C-C)	δ, μ	Na^+^ channels
		γ	Pacemaker channels
		κ	K^+^ channels
		ω	Ca^2+^ channels
	XIV (C-C-C-C)	α	Nicotinic acetylcholine receptors (nAChR)
		κ	K^+^ channels
	XXII (C-C-C-C-C-C-C-C)	ND	ND
B2	VIII (C-C-C-C-C-C-C-C-C-C)	α	Nicotinic acetylcholine receptors (nAChR)
		σ	Serotonin receptor
B3	XXIV (C-CC-C)	ND	ND
D	IV (CC-C-C-C-C)	α	Nicotinic acetylcholine receptors (nAChR)
		κ	K^+^ channels
		μ	Na^+^ channels
	XIV (C-C-C-C)	α	Nicotinic acetylcholine receptors (nAChR)
		κ	K^+^ channels
	XV (C-C-CC-C-C-C-C)	ND	ND
	XX (C-CC-C-CC-C-C-C-C)	α	Nicotinic acetylcholine receptors (nAChR)
	XXIV (C-CC-C)	ND	ND
	XXVIII (C-C-C-CC-C-C-C-C-C)	ND	ND
E	XXII (C-C-C-C-C-C-C-C)	ND	ND
G	XIII (C-C-C-CC-C-C-C)	ND	ND
I1	VI/VII (C-C-CC-C-C)	δ, μ	Na^+^ channels
		γ	Pacemaker channels
		κ	K^+^ channels
		ω	Ca^2+^ channels
	XI (C-C-CC-CC-C-C)	ι	Na^+^ channels
		κ	K^+^ channels
	XXI (CC-C-C-C-CC-C-C-C)	ND	ND
I2	VI/VII (C-C-CC-C-C)	δ, μ	Na^+^ channels
		γ	Pacemaker channels
		κ	K^+^ channels
		ω	Ca^2+^ channels
	XI (C-C-CC-CC-C-C)	ι	Na^+^ channels
		κ	K^+^ channels
	XII (C-C-C-C-CC-C-C)	ND	ND
	XIII (C-C-C-CC-C-C-C)	ND	ND
	XIV (C-C-C-C)	α	Nicotinic acetylcholine receptors (nAChR)
		κ	K^+^ channels
I3	VI/VII (C-C-CC-C-C)	δ, μ	Na^+^ channels
		γ	Pacemaker channels
		κ	K^+^ channels
		ω	Ca^2+^ channels
	XI (C-C-CC-CC-C-C)	ι	Na^+^ channels
		κ	K^+^ channels
J	XIV (C-C-C-C)	α	Nicotinic acetylcholine receptors (nAChR)
		κ	K^+^ channels
K	XXIII (C-C-C-CC-C)	ND	ND
L	XIV (C-C-C-C)	α	Nicotinic acetylcholine receptors (nAChR)
		κ	K^+^ channels
	XXIV (C-CC-C)	ND	ND
M	I (CC-C-C)	α	Nicotinic acetylcholine receptors (nAChR)
		ρ	Alpha1-adrenoceptors (GPCR)
	II (CCC-C-C-C)	α	Nicotinic acetylcholine receptors (nAChR)
	III (CC-C-C-CC)	α	Nicotinic acetylcholine receptors (nAChR)
		ι, μ	Na^+^ channels
		κ	K^+^ channels
	IV (CC-C-C-C-C)	α	Nicotinic acetylcholine receptors (nAChR)
		κ	K^+^ channels
		μ	Na^+^ channels
	VI/VII (C-C-CC-C-C)	δ, μ	Na^+^ channels
		γ	Pacemaker channels
		κ	K^+^ channels
		ω	Ca^2+^ channels
	IX (C-C-C-C-C-C)	ND	ND
	XIV (C-C-C-C)	α	Nicotinic acetylcholine receptors (nAChR)
		κ	K^+^ channels
	XVI (C-C-CC)	ND	ND
	XXXII (C-CC-C-C-C)	ND	ND
N	XV (C-C-CC-C-C-C-C)	ND	ND -
O1	I (CC-C-C)	α	Nicotinic acetylcholine receptors (nAChR)
		ρ	Alpha1-adrenoceptors (GPCR)
	VI/VII (C-C-CC-C-C)	δ, μ	Na^+^ channels
		γ	Pacemaker channels
		κ	K^+^ channels
		ω	Ca^2+^ channels
	IX (C-C-C-C-C-C)	ND	ND
	XII (C-C-C-C-CC-C-C)	ND	ND
	XIV (C-C-C-C)	α	Nicotinic acetylcholine receptors (nAChR)
		κ	K^+^ channels
	XVI (C-C-CC)	ND	ND
	XXIX (CCC-C-CC-C-C)	ND	ND
O2	I (CC-C-C)	α	Nicotinic acetylcholine receptors (nAChR)
		ρ	Alpha1-adrenoceptors (GPCR)
	VI/VII (C-C-CC-C-C)	δ, μ	Na^+^ channels
		γ	Pacemaker channels
		κ	K^+^ channels
		ω	Ca^2+^ channels
	XII (C-C-C-C-CC-C-C)	ND	ND
	XIV (C-C-C-C)	α	Nicotinic acetylcholine receptors (nAChR)
		κ	K^+^ channels
	XV (C-C-CC-C-C-C-C)	ND	ND
	XVI (C-C-CC)	ND	ND
O3	VI/VII (C-C-CC-C-C)	δ, μ	Na^+^ channels
		γ	Pacemaker channels
		κ	K^+^ channels
		ω	Ca^2+^ channels
	XVI (C-C-CC)	ND	ND
P	IX (C-C-C-C-C-C)	ND	ND
	XVI (C-C-CC)	ND	ND
Q	VI/VII (C-C-CC-C-C)	δ, μ	Na^+^ channels
		γ	Pacemaker channels
		κ	K^+^ channels
		ω	Ca^2+^ channels
	XVI (C-C-CC)	ND	ND
R	XIV (C-C-C-C)	α	Nicotinic acetylcholine receptors (nAChR)
		κ	K^+^ channels
S	VIII (C-C-C-C-C-C-C-C-C-C)	α	Nicotinic acetylcholine receptors (nAChR)
		σ	Serotonin receptor
	XXXIII (C-C-C-C-C-C-C-C-C-C-C-C)	ND	ND
T	I (CC-C-C)	α	Nicotinic acetylcholine receptors (nAChR)
		ρ	Alpha1-adrenoceptors (GPCR)
	V (CC-CC)	ε	Ca^2+^ channels or G protein receptors
		μ	Na^+^ channels
	X (CC-C[PO]C)	ε	Ca^2+^ channels or G protein receptors
	XVI (C-C-CC)	ND	ND
U	VI/VII (C-C-CC-C-C)	δ, μ	Na^+^ channels
		γ	Pacemaker channels
		κ	K^+^ channels
		ω	Ca^2+^ channels
V	XV (C-C-CC-C-C-C-C)	ND	ND
Y	VI/VII (C-C-CC-C-C)	δ, μ	Na^+^ channels
		γ	Pacemaker channels
		κ	K^+^ channels
		ω	Ca^2+^ channels
	XVII (C-C-CC-C-CC-C)	ND	ND
Undetermined	XVIII (C-C-CC-CC)	ND	ND
	XIX (C-C-C-CCC-C-C-C-C)	ND	ND
	XXV (C-C-C-C-CC)	ND	ND
	XXVI (C-C-C-C-CC-CC)	ND	ND
	XXVII (C-C-C-CCC-C-C)	ND	ND
	XXX (C-C-CCC-C-C-C-CC)	ND	ND

ND: not determined.

The diversity of mature conopeptides is further increased by post-translational modifications (PTMs) other than the formation of disulfide bonds, with the most common being C-terminal amidation, proline hydroxylation, and glutamate gamma-carboxylation [26]. It has been proposed that the unusually high frequency of PTMs in conopeptides is related to the pressure cone snails suffer to rapidly adapt their venoms to face environmental challenges [29]. It is, therefore, reasonable to infer that such modifications might have functional consequences, affecting the structure, potency, and even the selectivity of conopeptides, both naturally-occurring ones and synthesized analogs. In addition to PTMs, other mechanisms such as variable peptide processing and the maintenance of the propeptide region in the mature sequences of some conopeptides further increase their diversity [30].

By virtue of their specificity, diversity, and abundance, conopeptides are superb pharmacological tools and have great therapeutic potential. For example, the conotoxin ω-MVIIA, an N-type calcium channel blocker isolated from the venom of *Conus magus,* had its synthetic version - ziconotide (Prialt^®^) - approved by the Food and Drug Administration (FDA) for treatment of severe chronic pain [31]. Other conopeptides have been assessed as drug leads for a number of conditions that include pain, epilepsy, and diabetes, among others [32]. A few have reached the pre-clinical stage of development, for instance: RgIA4 (KCP-400), an analog of the α-RgIA from the venom of *Conus regius*, is an α9 α10 nAChR blocker that counteracts neuropathic pain [33]; and mini-Ins, a minimal insulin analog based on the insulin-like peptide found in the venom of *Conus geographus* that has been tested for type-I diabetes [34]. 

Considering the astounding diversity and potential of conopeptides, these molecules remain still very much unexplored [11, 35]. The advent of “omic” techniques furthered the identification of new conopeptides considerably, for their isolation and characterization through traditional chromatographic methods are laborious and slow. By combining transcriptomic screenings and modern proteomic techniques, the full content of both dissected and milked cone snail venoms can be determined up to the PTM profile of mature peptides [30]. Nevertheless, relatively few such molecules have been identified so far, and even fewer isolated and characterized, the vast majority from the venoms of Indo-Pacific species [26]. The larger distribution of cone snail species in this biogeographic region might account at least in part for this predominance, but other regions such as the Western Atlantic coast are no less relevant and are home to a considerable number of species [36]. 

**Figure 2.**
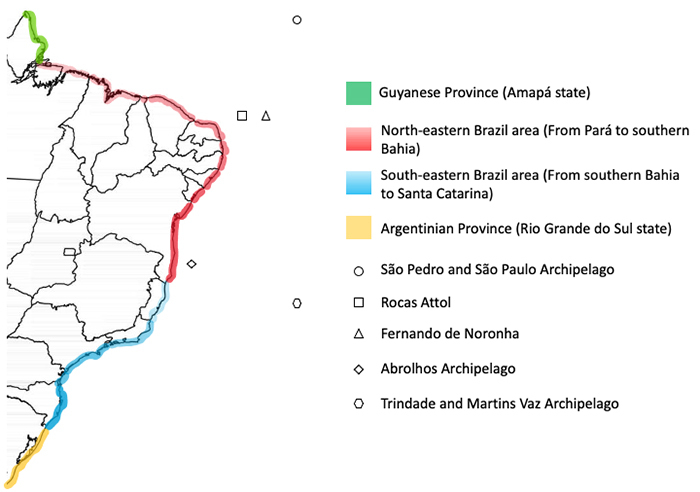
Biogeographical division of the Brazilian coast according to the distribution of marine prosobranch species from shallow waters proposed by Barroso et al. [37].

The Brazilian coast, for instance, is known for its privileged marine biodiversity, with diverse continental biogeographic regions and oceanic islands (Figure 2) [37] that favor the establishment of large populations of mollusks such as those of the genus *Conus*. To date, 31 species of cone snails have been described off the Brazilian coast, with 18 of them being endemic [38-40] (Table 3). Although, at first, these species were all classified as belonging to the genus *Conus*, it is now accepted that many of them actually belong to the genus *Conasprella* Thiele, 1929 [36]. Notwithstanding, these are all predatory, venomous snails. 

**Table 3:** *Conus* and *Conasprella* species found off the Brazilian coast.

Species - Accepted names	Biogeographical distribution (Brazilian coast)
*Conus* Linnaeus, 1758	🟩 🟥 🟦
*Conus (Attenuiconus) eversoni,* Petuch 1987*	🟥 □ △
*Conus (Brasiliconus) scopulorum,* Van Mol, Tursch & Kempf 1971*	🟥 ◯
*Conus (Chelyconus) ermineus,* Born 1778	🟩 🟥 🟦
*Conus (Dauciconus) cancellatus,* Hwass in Bruguière 1792	🟩 🟥 🟦 🟨
*Conus (Dauciconus) villepinii,* P. Fisher & Bernardi 1857	🟥 🟦 ⬡
*Conus (Dauciconus) riosi,* Petuch 1986*	🟥
*Conus (Dauciconus) hazinorum,* Petuch & Myers 2014*	◇
*Conus (Dauciconus) abrolhoensis,* Petuch 1987*	◇
*Conus (Dauciconus) cargilei,* Coltro 2004*	◇
*Conus (Dauciconus) pseudocardinalis,* Coltro 2004*	🟥
*Conus (Dauciconus) ziczac archetypus,* Crosse 1865^#^	🟥
*Conus (Dauciconus) fonsecai,* Petuch & Berschauer 2016*^#^	🟥
*Conus (Dauciconus) smoesi,* Petuch & Berschauer 2016*^#^	🟥
*Conus (Dauciconus) mariaodeteae,* Petuch & Berschauer 2014*^#^	🟥
*Conus (Dauciconus) tonisii,* Petuch & Berschauer 2014*^#^	🟦
*Conus (Lamniconus) petestimpsoni,* Petuch & Berschauer 2016*	🟥 🟦 🟨
*Conus (Lamniconus) clerii,* Reeve 1844	🟦
*Conus (Lamniconus) lemniscatus,* Reeve 1849	🟦 🟨
*Conus (Lamniconus) carcellesi,* Martins 1945	🟥 🟦
*Conus (Sandericonus) carioca,* Petuch 1986*	🟥 🟦 ◯ □ △
*Conus (Stephanoconus) regius,* Gmelin 1791	

*Conasprella* Thiele*,* 1929	
*Conasprella (Coltroconus) iansa,* Petuch 1979*	🟥
*Conasprella (Coltroconus) bodarti,* Coltro 2004*	🟥
*Conasprella (Coltroconus) delucai,* Coltro 2004*	🟥
*Conasprella (Coltroconus) schirrmeisteri,* Coltro 2004*	🟥
*Conasprella (Dalliconus) mazei,* Deshayes 1874	🟩 🟥 🟦 🟨
*Conasprella (Kohniconus) centurio,* Born 1778	🟥 🟦
*Conasprella (Ximeniconus) jaspidea,* Gmelin 1791	🟩 🟥 🟦
*Conasprella (Ximeniconus) pusio,* Hwass in Bruguière 1792	🟥 🟦
*Conasprella (Ximeniconus) mindana,* Hwass in Bruguière 1792	🟥 🟦
*Conasprella (Ximeniconus) henckesi,* Coltro 2004*	🟥

Biogeographical distribution colored according to the division depicted in Figure 2. Accepted names according to the World Register of Marine Species (WoRMS).

*Endemic species; ^#^also accepted as members of the genus *Poremskiconus*, Petuch 2013, now considered a synonym of the subgenus *Dauciconus*, Cotton 1945.

Probably because the vast majority of these animals are often restricted to small geographic areas, the venoms of cone snail species found off the Brazilian coast remain mostly unexplored [39]. As a result, only four of the species found in Brazil - *C. cancellatus, C. regius, C. villepinii, and C. ermineus* - have had their venoms partially characterized, and, although many conopeptides have been identified in these venoms, only a few have been isolated and characterized. This review focuses on the compilation of all knowledge regarding the biochemical and pharmacological properties described for the conopeptides and other toxic components identified in the venoms of the aforementioned species thus far. Searches were performed on PubMed, ConoServer, and WoRMS databases, using either the names of the species - for PubMed and WoRMS - or the names of the toxins - for ConoServer. The last searches were conducted on August 13, 2022.

## *Conus cancellatus*

A subspecies from the vermivorous *C. cancellatus* - known as *C. cancellatus cancellatus* or *C. austini* - found in the Caribbean Sea, Colombia, Gulf of Mexico, Venezuela, and off the Brazilian coast, had six peptides identified in its venom so far (Table 4). Of these, four are conotoxins that were isolated by reverse-phase high performance liquid chromatography (RP-HPLC) and had their primary sequences and molecular mass determined by Edman degradation and matrix-assisted laser desorption/ionization - time of flight (MALDI-ToF), respectively. 

**Table 4.** Conopeptides identified in the venom of *Conus cancellatus*.

Name (protein card)	MM	Sequence	G. Sf	Cys F	References
γ-AsVIIA (P0147)*	3282.61	T**C**KQKGEG**C**SLDV(Gla)**CC**SSS**C**KPGGPLFDFD**C**	O2	VI/VII	[41]
κ-like-AsXIVA (P02828)*	2886.35	GGVGR**C**IYN**C**MNSGGGLNFIQ**C**KTM**C**Y	ND	XIV	[42]
κ-like-AsXIVB (P02830)*	3154.51	WDVDQ**C**IYY**C**LNGVVGYSYTE**C**QTM**C**T	ND	XIV	[41]
As25a/b (P05524)*	2678.05	**C**K**C**(O)S**C**NFNDVTEN**C**K**CC**IFRQ(O)(nh2)	ND	XXV	[43]
As1a/b (P08700)^#^	1632.08	RIKKPIFIAFPRF(nh2)	Conorfamide	NA	[44]
As2a/b (P08701)^#^	1660.10	RIRKPIFIAFPRF(nh2)	Conorfamide	NA	[44]

*Conotoxins and ^#^disulfide-poor conopeptides. MM: molecular mass; G. Sf: gene superfamily, Cys F: cysteine framework according to ConoServer [26]. (Gla): gamma carboxylic glutamic acid; (O): 4-hydroxyproline; (nh2): C-terminal amidation; ND: not determined; NA: not applicable. All sequences were determined at protein level.

The first conotoxin isolated from the venom of *C. cancellatus* is a 31-residue peptide named AsVIIA [41]. It was classified as a γ-conotoxin from the O2 superfamily with a VI/VII cysteine framework. This somewhat rare pharmacological family of conotoxins, which have a conserved -(Gla)CCS- motif in their primary structures, modulates neuronal pacemaker cation currents in mollusks [45]. Toxins belonging to this family have been identified in three molluscivorous [46-48] and one vermivorous species [49] apart from *C. cancellatus*. Similar to other γ-conotoxins such as TxVIIA from *Conus textile* [46] and γ-PnVIIA, from *C. pennaceus* [49], AsVIIA induced foot shrinking in the freshwater snail *Pomacea paludosa* after intramuscular (i.m.) injection [41]. This biological activity towards mollusks and the structural similarities between AsVIIA and other γ-conotoxins suggest that they share the same unidentified pacemaker channels as possible targets [41].

Another two conotoxins were isolated from the *C. cancellatus* venom by the same research group - AsXIVA and AsXIVB. These 27-residue peptides share about 40% identity and belong to cysteine framework XIV, although their gene superfamily and pharmacological family remain unknown [42]. AsXIVA exhibited sequence similarity with VilXIVA, a conotoxin from *C. villepinii* whose 3D structure resembles that of K^+^ channel blockers [50]. In addition, AsXIVA may have two Lys/Tyr dyads, a motif identified in K^+^ channel blockers [51, 52], suggesting that this conotoxin might also target these channels. On the other hand, AsXIVB exhibited sequence similarity with FlfXIVB, a conotoxin from the vermivorous *Conus floridanus floridensis* (*Conus anabathrum*) [50], which suggests that both have the same - yet undetermined - pharmacological target. Both AsXIVA and AsXIVB increased scratching and grooming activities in mice after intracranial (i.c.) injection, although the latter provoked a more noticeable and durable effect, inducing also body and rear limbs extension and tail curling [42].

A 23-amino acid conotoxin named As25a and its post-translationally modified variant As25b, in which Pro4 and Pro23 are hydroxylated, have been also isolated from the venom of *C. cancellatus* [43]. At first, this conotoxin and its modified counterpart had been named As24a and As24b, but they were renamed because of a nomenclature switch of their cysteine framework from XXIV to XXV. Although As25a has not yet been classified into any conotoxin superfamily, it has some sequence similarity with conotoxins from the M-superfamily, which can target Na^+^ channels, K^+^ channels, pacemaker channels, and nAChRs [53]. As25a (i.c.) induced hind limb paralysis and death in mice [43]. 

In addition to the aforementioned conotoxins, two conopeptides from the conorfamide family were isolated from the *C. cancellatus* venom: conorfamides As1a and As2a and their non-amidated counterparts, As1b and As2b. They all had their molecular masses determined by MALDI-ToF and were sequenced by mass spectrometry (MS)-based *de novo* sequencing, followed by confirmation through Edman degradation. These nearly identical 13-residue peptides were isolated through an activity-guided fractionation assay assessing α7 nAChR activity and their sequences suggested an association with the conorfamide family, whose targets include also acid-sensing ion channels (ASIC) [44, 45]. All four peptides inhibited α7- and muscle-type nAChRs, but the amidated isoforms were more potent than the non-amidated ones. In addition, As1a and As2a were more potent against α7 when compared with muscle-type nAChRs, and their effects on the former were slowly reversible while those on the latter were quickly reversible [44]. As1a and As2a inhibited the desensitization of rat ASIC1a and, to a lesser degree, ASIC3 channels, which resulted in a sustained current at low pH. The non-amidated variants As1b and As2b were mostly ineffective, except for a discrete inhibition of ASIC1a by As1b [44]. These results add to the evidence pointing to a crucial role of PTMs in the activity of conopeptides. 

## *Conus regius*

*C. regius* is a vermivorous species found in the Western Atlantic (north Florida), Gulf of Mexico, and off the Brazilian coast. Thirty-three conotoxins have been identified in the venom of this species to date (Table 5).

**Table 5.** Conotoxins identified in the venom of *Conus regius.*

Name (protein card)	MM	Sequence	G. Sf	Cys F	References
Rg9.1 (P01509)^Δ^	4021.66	F**C**GQA**C**SSVK**C**PKK**C**F**C**HPEEKV**C**YREMRTKERD	P	IX	[54]
RgXIA (P01508)^ρ^	4695.30	**C**QAYGES**CC**SAVVR**CC**DPNAV**CC**QYPEDAV**C**VTRGY**C**RPPATVLT	I1	XI	[55]
α-RgIA (P02585)^Δ^	1570.79	G**CC**SDPR**C**RYR**C**R	A	I	[56, 57]
α-Reg1b/c (P00028)^ρ^	1347.51	G**CC**SD(O)R**C**KHQ**C**(nh2)	A	I	[57]
α-Reg1d (P00029)^ρ^	1332.50	G**CC**SDPR**C**KHE**C**(nh2)	A	I	[57]
α-Reg1f (P00031)^ρ^	1569.84	DY**CC**RR(O)(O)**C**TLI**C**(nh2)	A	I	[57]
α-RegIIA (P00032)^ρ^	1663.88	G**CC**SHPA**C**NVNNPHI**C**(nh2)	A	I	[57]
α-RgIB (P05941)^ρ^	2700.98	TWEE**CC**KNPG**C**RNNHVDR**C**RGQV	ND	I	[58]
Reg3a (P08570)^ρ^	1814.97	G**CC**(O)(O)QW**C**G(O)D**C**TS(O)**CC**	M	III	[57, 59]
Reg3b (P07504)^ρ^	1669.99	**CC**TAL**C**SRYHCLP**CC**	M	III	[59]
Reg3c (P08572)^ρ^	1787.09	**CC**AF(O)QW**C**GAG**C**IV(O)**CC**	M	III	[59]
Reg3d (P08573)^ρ^	1767.95	L**CC**(O)(O)Q(O)**C**G(O)D**C**AS(O)**CC**	M	III	[59]
Reg3e (P08533)^ρ^	1738.17	K**CC**MRPI**C**T**C(**O)**CC**IGP	M	III	[57, 59]
Reg3f (P08536)^ρ^	1821.20	G**CC**PFPA**C**TTHII**C**R**CC**(nh2)	M	III	[57, 59]
Reg3g (P08560)^ρ^	1699.09	**CC**MAL**C**SRYH**C**LP**CC**(nh2)	M	III	[57, 59]
Reg3h (P08538)^ρ^	1209.42	G**CC**S(O)WN**C**IQLRA**C**(O)**CC**(O)N(nh2)	M	III	[57, 59]
Reg3i (P0DPJ6)^ρ^	1831.19	**CC**AIRL**C**NVYLCGS**CC**(O)	M	III	[59]
Reg3j (P08561)^ρ^	1827.12	G**CC**S(O)WN**C**IQLRA**C**G**CC**	M	III	[59]
Reg3k (P08534)^ρ^	1767.27	K**CC**MRPI**C**M**C**(O)**CC**IGP(nh2)	M	III	[59]
Reg3l (P08541)^ρ^	1982.42	R**CC**PMPG**C**FAGPF**C**P**CC**PV	M	III	[57, 59]
Reg3m (P08543)^ρ^	ND	IVR**CC**SAT**C**K(X)S**C**V**CC**F	M	III	[57, 59]
Reg3.5 (P08544)^Δ^	1399.71	**CC**MRPV**C**T**C**P**CC**S	M	III	[59]
Reg3.6 (P08546)^Δ^	1899.31	G**CC**PYPK**C**IHVTF**C**K**CC**	M	III	[59]
Reg3.7 (P08548)^Δ^	1776.06	**CC**PYPS**C**IDIPF**C**D**CC**	M	III	[59]
Reg3.8 (P08550)^Δ^	1825.20	**CC**PFPM**C**YQVPH**C**P**CC**	M	III	[59]
Reg3.9 (P08552)^Δ^	1737.08	**CC**TIWN**C**VQLPGCP**CC**	M	III	[59]
Reg3.10 (P08554)^Δ^	2132.55	**CC**HPNL**C**IGRSALGRK**C**T**CC**	M	III	[59]
Reg3.11(P08556)^Δ^	1592.77	**CC**RIEF**C**DED**C**G**CC**	M	III	[59]
Reg3.12 (P08558)^Δ^	1490.63	**CC**EVGW**C**DSG**C**E**CC**	M	III	[59]
Reg3.14 (P08562)^Δ^	1828.15	**CC**NWPR**C**NVYL**C**GP**CC**	M	III	[59]
Reg3.15 (P08564)^Δ^	1507.84	**CC**PIQG**C**ILG**C**TP**CC**	M	III	[59]
Reg3.16 (P08566)^Δ^	1703.95	**CC**YEEE**C**PPS**C**KL**CC**	M	III	[59]
Reg3.17 (P08568)^Δ^	1450.70	**CC**TGQ**C**HI**C**WP**CC**	M	III	[59]

ρ: sequences determined at protein level; Δ: sequences deduced from precursor. MM: molecular mass; G. Sf: gene superfamily; Cys F: cysteine framework according to ConoServer [26]; O: 4-hydroxyproline; nh2: C-terminal amidation; X: unidentified amino acid; ND: not determined.

The first conotoxin identified in the venom of *C. regius* is a 34-residue peptide named Rg9.1, whose sequence was deduced from its precursor cDNA [54]. Although its pharmacological target remains unknown, Rg9.1 was classified as a member of the P superfamily, cysteine framework IX, which harbors TxIXA, a conotoxin from the molluscivorous *C. textile* that causes spasms in mice [60]. 

Soon after, the same research group identified a 44-residue peptide that belongs the I1 superfamily, cysteine framework XI, named RgXIA [55]. This conotoxin was isolated through RP-HPLC and had its molecular mass and sequence determined through MALDI-ToF and *de novo* sequencing, respectively. Although its biological activity is still under investigation, RgXIA is homologous to BtX [61] and ViTx [62], κ-conotoxins from *Conus betulinus* and *Conus virgo,* respectively*,* that modulate vertebrate K^+^ channels.

The best-studied conotoxins identified in the venom of *C. regius* are the α-conotoxins RgIA, RegIIA, and RgIB, which target nAChRs. As potent modulators of different neuronal and muscular isoforms of these receptors [63], α-conotoxins are useful tools in the investigation of chronic pain and inflammation, having been extensively employed in these fronts.

**Figure 3.**
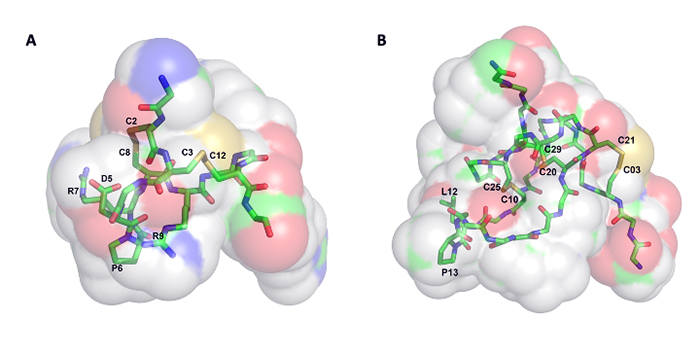
Three-dimensional structures of conotoxins. **(A)** Three-dimensional structure of RgIA, an α-conotoxin from the *Conus regius* venom, classified into A superfamily, cysteine framework I, and fold A. The residues involved in the interaction with α9α10 nAChRs - D5, P6, and R7, and the residue that determines selectivity - R9, are highlighted. **(B)** Three-dimensional structure of the cis-isomer of EVIA, a δ-conotoxin from *Conus ermineus* venom, classified into O1 superfamily, cysteine framework VI/VII, and fold C. The L12-P13 residues, which are connected by a peptide bond that shows a 1:1 cis/trans isomerism, are highlighted. Both structures are represented as stick models with surface models on the background. The images were produced using *PyMOL* [66], from the models 2JUS for RgIA [64] and 1GIZ for EVIA [67] deposited on the Protein Data Bank (PDB - https://www.rcsb.org/).

RgIA is an α-conotoxin from the A superfamily, cysteine framework I, and loop class 4/3. The sequence of the mature 13-residue peptide was first deduced from the nucleotide sequence [56], and later confirmed by Edman degradation when RgIA was isolated by RP-HPLC along four other similar α4/3 conotoxins - Reg1b/c, Reg1d, Reg1e, and Reg1f - in a study focused on the post-translational hydroxylation of conopeptides [57]. It is now accepted that RgIA and Reg1e are most likely the same peptide, as the only difference in their mature sequences is the absence of an arginine residue at the C-terminus in the latter. Analysis by nuclear magnetic resonance (NMR) of the 3D structure of RgIA (Figure 3A) and a few synthesized analogues revealed that this conotoxin assumes a globular (fold A), two-loop backbone architecture, with the residues Asp5, Pro6, and Arg7 in the loop 1 being important for the interaction between the toxin and α9α10 nAChRs, although selectivity to this receptor is actually determined by the Arg9 in the loop 2 [64, 65].

RgIA showed a dose-dependent antinociceptive action in rats, as it increased the paw withdrawal threshold that had been reduced by chronic constriction injury of the sciatic nerve [68]. Moreover, it affected the peripheral immune response to nerve injury by reducing the number of choline acetyltransferase-immunoreactive cells, ED1-immunoreactive macrophages, and CD2-immunoreactive T cells at the injury site [68]. This same neuropathic injury model was used in another study that investigated the analgesic effect of RgIA [69]. It was observed that the repeated administration (i.m.) of this conotoxin into the ipsilateral paw significantly prevented the development of pain hypersensitivity in injured rats, increasing their pain threshold, relieving the postural imbalance caused by the injury in up to 80%, and protecting the ipsilateral sciatic nerve against morphological derangements [69]. In addition, the treatment reduced the edema and inflammatory infiltrate - particularly CD86+ cells - observed in the constricted nerve, prevented the reduction in the somatic area of L4-L5 dorsal root ganglia (DRG) neurons induced by the constriction injury to the sciatic nerve, and inhibited the activation of astrocytes and microglia in the dorsal horn of the spinal cord [69]. RgIA was also effective against chemotherapy-induced neuropathy, as the concomitant intraperitoneal (i.p.) administration of this toxin induced both analgesic and neuroprotective effects in rats treated with oxaliplatin (i.m.) - a drug often employed in the treatment of colorectal cancer - for three weeks [70]. RgIA reduced mechanical hypersensitivity and the sensitivity to cold noxious stimuli in these animals, in addition to partially preventing the morphological changes induced by this drug in L4-L5 DRG neurons [70]. It has been recently shown that RgIA reduces the damage caused by dextran sodium sulfate-induced colitis in mice: the subcutaneous (s.c.) injection of this conotoxin reversed the severity of the disease in various aspects, including a reduction in the colonic levels of TNF-α, which suggests that α9α10 nAChRs may be involved in pro-inflammatory mechanisms that take place in colitis [71]. 

Besides modulating nicotinic receptors, RgIA has other targets: it inhibited high-voltage-activated (HVA) Ca^2+^ currents in rat DRG neurons via activation of γ-aminobutyric acid (GABA)B G-protein coupled receptors and Ca_v_2.2 (N-type) channels expressed in baclofen-sensitive *Xenopus* oocytes [72]. The participation of GABAB receptors in the modulation of HVA Ca^2+^ channels by RgIA was further confirmed when the activity of this conotoxin was significantly reduced in GABAB-knockdown DRG neurons and also when RgIA failed to inhibit Ca_v_2.2 currents in HEK cells in the absence of the two subunits of GABAB [73]. 

The α-conotoxin RegIIA was isolated along RgIA in the same RP-HPLC process [57, 74]. Although it also belongs to the A superfamily and cysteine framework I, this 16-residue peptide is classified into loop class 4/7 instead. RegIIA is nearly identical to OmIA, an α-conotoxin from the molluscivorous *Conus omaria*, the only difference being that the latter has an extra glycine residue at the C-terminal position [74]. It is also highly homologous to GIC [75] and GID [76], two α4/7 conotoxins isolated from the venom of the fish-hunting *C. geographus* [57]. Unlike its counterparts, RegIIA was found to be primarily a potent antagonist of α3β4 nAChRs, and, to a lesser degree, of α3β2 and α7 nAChRs, which are the usual targets of α4/7 conotoxins [74]. The 3D structure of RegIIA obtained by NMR revealed that, like RgIA, it belongs to the classical fold A category, with a distribution of charged, polar, and hydrophobic residues in its surface that could account for its unique selectivity [74]. The effect of RegIIA on both α3β4 and α7 receptors was completely abolished when the asparagine residue at position 9 in the loop II was replaced by alanine [77]. More importantly, a potent α3β4 nAChRs-selective antagonist ([N^11^A,N^12^A]RegIIA) was synthesized by replacing the asparagine residues at positions 11 and 12 by alanine, shedding some light into the differences between the interaction of RegIIA with its various targets [77]. There is also a difference regarding the interaction between RegIIA and nAChR from different species, but this species-specificity does not encompass all the subtypes targeted by this toxin [78]. For instance, RegIIA and the analogue [N^11^A,N^12^A]RegIIA blocked human and rat α3β4 nAChRs equally. On the other hand, the native toxin completely blocked the current evoked by the rat α3β2 subtype while only reducing that from the human α3β2 subtype [78]. Surprisingly, this difference in selectivity was associated to the amino acid residue in the position 198 of the α3 subunit: a glutamine in the rat subtype and a proline and the human subtype [78]. As the α3 subunit is shared between the α3β4 and α3β2 subtypes, it stands to reason that the differences regarding the species-specificity shown by RegIIA towards these subtypes is strongly influenced by the type of β subunit interacting with α3.

RgIB, yet another nAChR-blocker, completes the list of α-conotoxins already described in the venom of *C. regius* thus far [58]. It was identified by MALDI-ToF following the RP-HPLC fractionation of the crude venom and sequenced through electrospray-ionization quadrupole time-of-flight (ESI-Q-ToF) mass spectrometry. This 23-residue toxin is the largest conotoxin identified in this venom, having been classified as a member of the cysteine framework I group, although its loop class and gene superfamily remain unknown. At high doses, the injection (i.c.) of RgIB in mice induced hyperactivity, while lower doses led to respiratory difficulties. In addition, α-RgIB partially blocked - in an irreversible manner - the slow-desensitizing ionic currents from differentiated PC12 neurons, which express α3β4 and/or α3β4α5 nAChRs [58].

Last but not least, a large group of mini-M conotoxins - a subclass of M-conotoxins that have either one (M1), two (M2) or three (M3) residues in the third loop - was identified in the venom of *C. regius* [57, 59]. Thirteen of them - Reg3a-m - were isolated through RP-HPLC and sequenced by Edman degradation [57, 59]. Although they all belong to cysteine framework III, there is very little homology between their primary sequences, which fall into eight different loop classes: 3/3/1, 3/4/2, 4/1/1, 4/2/3, 4/3/3, 4/4/2, 4/5/1, and 5/3/3. In addition, these toxins present various degrees of PTMs, which, combined to the differences in their sequences, suggests they modulate different, yet undetermined targets. The structure of Reg3b, solved by NMR, revealed that the toxin assumes a compact, globular fold that comprises a series of turns [59]. The same research group identified 12 other mini-M conotoxins in the transcriptome of the *C. regius* venom, named Reg3.5-12, and 3.14-17 [59]. Their sequences were proved as diverse as those of their isolated counterparts, adding the loop classes 3/2/2, 4/3/1, 4/3/2, and 4/9/1 to those already described for the hypervariable *C. regius* mini-M conotoxins. 

## *Conus villepinii*

This vermivorous species is found along the Brazilian coast, Florida, West Indies, and Uruguay. It had 12 toxins identified in its venom to date (Table 6).

**Table 6.** Conopeptides identified in the venom of *Conus villepinii*.

Name (Protein card)	MM	Sequence	G. Sf	Cys F	References
VilXIVA (P01619)*^ρ^	2873.35	GGLGR**C**IYN**C**MNSGGGLSFIQ**C**KTM**C**Y	R	XIV	[50]
VilXIVB (P08492)*^Δ^	3160.53	WDVDQ**C**MYY**C**LTGVVGYSYTE**C**ETM**C**T	R	XIV	[79]
VilXIVC (P08495) *^Δ^	3159.54	WDVDQ**C**MYY**C**LTGVVGYSYTE**C**QTM**C**T	R	XIV	[79]
Vil14.8*^Δ^§	ND	GGLGR**C**IYN**C**MNSGGGLSFIQ**C**KTM**C**Y	R	XIV	[79]
Vil14.9*^Δ^§	ND	GGVEQ**C**IYN**C**LTGYIGRSYIQ**C**KTM**C**T	R	XIV	[79]
Vil14.10*^Δ^§	ND	GGVEQ**C**IYN**C**LTGYIGGSYIQ**C**KTM**C**T	R	XIV	[79]
Vil14.11*^Δ^§	ND	WDVDQ**C**MYY**C**LTGVLEYSYTE**C**ETM**C**T	R	XIV	[79]
Vil14.12*^Δ^§	ND	WDVDQ**C**IYY**C**LTGVVGYSYTE**C**ETM**C**T	R	XIV	[79]
γ-conopressin-vil (P01307)^#ρ^	1018.12	**C**LIQD**C**P(Gla)G(nh2)	Conopressins	NA	[80]
conoCAP-Vila (P07533)^#ρ^	1148.27	PF**C**NSFG**C**YN(nh2)	conoCAPs	NA	[81]
conoCAP-Vilb (P07532)^#Δ^	1001.14	VF**C**NGFTG**C**G(nh2)	conoCAPs	NA	[81]
conoCAP-Vilc (P07534)^# Δ^	1143.30	LF**C**NGYGG**C**RG(nh2)	conoCAPs	NA	[81]

*Conotoxins and ^#^disulfide-poor conopeptides. ρ: sequences determined at protein level; Δ: sequences deduced from precursor. MM: molecular mass; G. Sf: gene superfamily; Cys F: cysteine framework according to ConoServer [26]. §: no ConoServer protein card, name and sequence obtained from the publication itself [77]. (Gla): gamma carboxylic glutamic acid; (nh2): C-terminal amidation; ND: not determined; NA: not applicable.

The first conotoxin identified in the venom of *C. villepinii* is a 27-residue peptide named VilXIVA, isolated by size exclusion (SE) followed by RP-HPLC and sequenced by Edman degradation [50]. Its four cysteine residues are arranged in a previously unreported framework, then classified as framework XIV. This same framework was identified in FlfXIVA and FlfXIVB, conotoxins from the venom of *C. anabathrum* whose cDNA precursor revealed a signal sequence that defined a new gene superfamily, then named R superfamily [79]. Through this conserved signal sequence, seven additional R/XIV conotoxins were cloned from the cDNA of *C. villepinii* venom: VilXIVB, VilXIVC, and Vil14.8-12 [79]. In addition to belonging to the same superfamily and cysteine framework, the aforementioned *C. villepinii* conotoxins share the same loop class, 3/11/3. Three of them - vil14.8-10 - share high sequence identity with VilXIVA, while the other ones display high similarity with the R-conotoxins from *C. anabathrum* venom. A molecular model of VilXIVA based on NMR data revealed that this conotoxin has a well-defined three-dimensional structure in solution, which resembles that of K^+^ channel blockers isolated from scorpion venoms [50]. Nevertheless, framework XIV toxins belonging to other gene superfamilies can have different targets; for instance, LtXIVA - an αL-conotoxin from the vermivorous *Conus literatus* - inhibited neuronal nAChRs [82]. The actual target of the *C. villepinii* R-toxins remains to be determined. 

In addition to the aforementioned conotoxins, single-disulfide conopeptides were identified in the *C. villepinii* venom. For instance, a 9-residue vasopressin/oxytocin-like peptide, named γ-conopressin-vil, was isolated from this venom by SE- and RP-HPLC and sequenced through Edman degradation [80]. The eighth residue in this conopressin is a gamma-carboxyglutamate, a unique feature that distinguishes γ-conopressin-vil from other conopressins described so far [12, 83, 84]. NMR spectroscopy data revealed that γ-conopressin-vil goes through Ca^2+^-mediated conformational changes as a result of the gamma-carboxyglutamate in its structure. The net charge of this residue (-2) could affect the electrostatic surface of γ-conopressin-vil, and, consequently, the way it binds to its yet undetermined target [80]. 

Another three disulfide-poor conopeptides were identified in the venom of *C. villepinii*, all crustacean cardioactive peptide (CCAP)-like toxins named conoCAP-Vila, conoCAP-Vilb, and conoCAP-Vilc [81]. The 10-residue conoCAP-Vila was isolated through SE- and RP-HPLC and sequenced by Edman degradation, while its counterparts were identified through cloning of the multi-peptide conoCAP precursor. ConoCAP-Vila is a hydrophobic peptide with three aromatic residues that showed high sequence identity with other known CAPs. In addition to decreasing the heart rate and causing arrhythmia in *Drosophila melanogaster* larvae, ConoCAP-Vila decreased the mean arterial blood pressure and heart rate of rats [79]. It also promoted a time-dependent and irreversible decrease in the amplitude of systolic Ca^2+^ transients and in the contractile activity of rat cardiac myocytes, although it had no effect on L-type Ca^2+^ currents [81].

## *Conus ermineus*

*Conus ermineus* is the only piscivorous species described off the Brazilian coast to date, being also found off the Caribbean, the northern coast of South America, and off the African coast. The venom from this species has been fairly well-explored, and a large number of molecules were identified, mostly through transcriptomic analysis. Amongst all the conopeptides reported to be present in the venom of this species, 24 have had their sequences published and are listed in Table 7. 

**Table 7.** Conopeptides identified in the venom of *Conus ermineus*.

Name (protein card)	MM	Sequence	G. Sf	Cys F.	References
α-EI (P00050)*^ρ^	2093.36	RD(O)**CC**YHPT**C**NMSNPQI**C**(nh2)	A	I	[85]
α-EIIA (P04069)*^ρ^	1774.03	(Z)T(O)G**CC**WNPA**C**VKNR**C**(nh2)	A	I	[86]
α-EIIB (P07538)*^ρ^	1754.99	(Z)T(O)G**CC**WHPA**C**GKNR**C**(nh2)	A	I	[87]
E1.1(P03001)*^Δ^	1787.96	DP**CC**SNPA**C**NVNNPQI**C**	A	I	[88]
E1.2(P08455)*^Δ^	1757.00	QTPG**CC**WHPA**C**GKNR**C**	A	I	[88]
E1.3(P08471)*^Δ^	1999.14	DD**CC**PDPS**C**RQNHPEL**C**A	A	I	[88]
αA-EIVA (P01635)*^ρ^	3096.42	G**CC**GPY(O)NAA**C**H(O)**C**G**C**KVGR(O)(O)Y**C**DR(O)SGG(nh2)	A	IV	[89]
αA-EIVB (P01740)*^ρ^	3100.40	G**CC**GKY(O)NAA**C**H(O)**C**G**C**TVGR(O)(O)Y**C**DR(O)SGG(nh2)	A	IV	[89]
E4.1(P08469)*^Δ^	4055.75	D**CC**GVKLDM**C**HP**C**L**C**NNS**C**KQGQGKKRVWEMMKATD	A	IV	[88]
δ-EVIA (P01561)*^ρ^	3287.85	DD**C**IK(O)YGF**C**SLPILKNGL**CC**SGA**C**VGV**C**ADL(nh2)	O1	VI/VII	[90]
δ-EVIB (P01575)*^Δ^	2972.48	EA**C**Y(O)(O)GTF**C**GIK(O)GL**CC**SEL**C**LPAV**C**VG(nh2)	O1	VI/VII	[91]
E6.1(P03273)*^Δ^	3618.22	ATSNRP**C**KPKGRK**C**FPHQKD**CC**NKT**C**TRSK**C**P	O1	VI/VII	[88]
E6.2 (P03274)*^Δ^	2777.14	Q**C**TPHGGS**C**GLVST**CC**GR**C**SVPRNK**C**E	O1	VI/VII	[88]
E5.1(P08457)*^Δ^	1423.70	D**CC**PEKMW**CC**PL	T	V	[88]
E5.2(P08461)*^Δ^	2756.09	TEHFPLMIWVD**CC**PAYD**CC**VPDSD	T	V	[88]
E5.3(P08463)*^Δ^	1416.61	GP**CC**FSNPY**CC**NL	T	V	[88]
E20.1 (P08483)*^Δ^	5070.71	AVIAT**C**HPNYPGSPWGR**CC**TTKM**C**GSV**CC**NYAH**C**S**C**VYHSDMGDG**C**S**C**	D	XX	[88]
E22.1 (P08485)*^Δ^	8255.59	WPRLTDSD**C**ELGRNMHIT**C**KQLDQ**C**GVIEKKDGQLT**C**KLR**C**K**C**KPGKR**C**LRKENIDWSDITTRIYH**C**PWP	E	XXII	[88]
Conantokin-E1 (P03534)^#Δ^	3139.19	GE(Gla)(Gla)HSKYQ(Gla)**C**LR(Gla)IRVNNVQQ(Gla)**C**	Conantokin	NA	[92]
Conantokin-E2 (P08574)^#Δ^	2034.16	SSEEDIELIEALEESGKR	Conantokin	NA	[86]
Conkunitzin-E3 (P08477)^#Δ^	8944.91	DTVPGLSALTVDDDTVPDV**C**RQPLEVGP**C**KAAYPRYYYNHASDT**C**QLFYYGG**C**NGNENRFEDFSG**C**LFT**C**IYPWMAALGY	Conkunitzin	NA	[88]
Conkunitzin-E4 (P08479)^#Δ^	4318.87	**C**HLPPETGM**C**RAYIPMHFYNATLGR**C**QGFIYGG**C**NGNDN	Conkunitzin	NA	[88]
Conkunitzin-E5 (P08481)^#Δ^	9869.11	**CC**HPP**C**EYGER**C**VSANRRERRHRHNNV**C**VPVR**C**LFQARRGR**C**LNFDRRYHFNTLTMS**C**TRVHTGA**C**YGRNNRFSSSEN**C**ELT**C**AP	Conkunitzin	NA	[88]
Con-ikot-ikot-E1 (P08474)^#Δ^	8043.11	DD**CC**IGNTYG**C**LKRRPGQEHEQVMP**C**KHEATIR**C**PGSDIDG**CC**PGYAT**C**MSIFAKDNLIPAHYH**C**EKRP**C**YT	Con-ikot-ikot	NA	[88]

*Conotoxins and ^#^disulfide-poor conopeptides. MM: molecular mass; G. Sf: gene superfamily; Cys F: cysteine framework according to ConoServer [26]. (Gla): gamma carboxylic glutamic acid; (Z): pyroglutamic acid; (O): 4-hydroxyproline; (nh2): C-terminal amidation; ND: not determined; NA: not applicable; ρ: sequences determined at protein level; Δ: sequences deduced from precursor.

A few conotoxins from the A superfamily were isolated from the venom *of C. ermineus*. The first one to be described was EI, an 18-aminoacid α4/7-conotoxin with four cysteine residues arranged into framework I, isolated from the milked venom of this species through RP-HPLC [83]. As expected, the injection (i.m.) of nanomolar concentrations of EI into fish led to paralysis; in mice, it caused muscle weakness, which eventually evolved to paralysis and death [83]. Binding assays revealed that this α-conotoxin selectively binds the α/δ site of *Torpedo* nAChRs, and, although this is also its preferred site in mammalian receptors, EI can also bind the α/γ site in the latter [85]. It has been recently shown that: (i) the point mutations of residues His7, Pro8, Met12, and Pro15 into alanine significantly reduced the effect of EI on α1β1δε nAChRs; (ii) the replacement of a critical serine residue at position 13 by alanine increased the potency of the toxin against this muscle-type nAChR while reducing its effect on the neuronal α3β2 and α3β4 subtypes; and (iii) the potency against α1β1δε nAChRs was related to the Arg1-Asn2-Hyp3 residues at the N-terminus of the toxin, as the deletion of these residues in the analogue ^△1-3^EI caused total loss of effect on this subtype [93].

Another α-conotoxin from the A superfamily, named EIIA, was identified in the venom of *C. ermineus* by an MS-based ‘fishing’ technique, using common features of α-conotoxins as hooks [84]. This 16-residue peptide, which belongs to cysteine framework I and loop class 4/4, is highly homologous to PIB, an α-conotoxin from the fish-hunting, Eastern Pacific species *Conus purpurascens* that selectively blocks muscle-type α1β1δε nAChRs [94].

Similarly, the synthetic EIIA was found to be highly selective for the muscle-type nAChR present in *Torpedo* membranes, distinguishing between its two acetylcholine binding sites [86]. A nearly identical isoform of EIIA, named EIIB, was identified in this venom through affinity-selection mass spectrometry, and they differ from each other only in the residues H8N and G12V [87]. As expected, radioligand binding assays revealed that a synthetic EIIB homologue bound with high affinity to *Torpedo* nAChRs [87].

Two αA-conotoxins named EIVA and EIVB, which also belong to the A superfamily, were isolated from the milked venom of *C. ermineus* by RP-HPLC [89]. These nearly identical 30-residue peptides were classified into cysteine framework IV, loop class 7/2/1/7, and fold I [11], being homologous to the αA-conotoxin PIVA from *C. purpurascens*, an antagonist of both α/δ and α/g sites in muscle-type nAChRs from mice [95]. The functional characterization of the synthetic EIVA through electrophysiology and binding assays in *Torpedo* and mouse nAChRs revealed that it also interacts with both α/δ and α/γ sites, though with higher affinity than PIVA [89, 96]. The higher potency of EIVA can be the result of structural differences between this toxin and its *C. purpurascens* counterpart: not only EIVA has four additional residues in its C-terminus but also a more hydrophobic, protruding region in which the residue at position 18 is a valine instead of the aspartic acid present in PIVA, as revealed by the 3D structures of both toxins solved by NMR [96].

Members from the O1 superfamily have also been found in the venom of *C. ermineus*. A δ-conotoxin named EVIA was isolated from this venom through RP-HPLC in multiple steps and sequenced by Edman degradation [90]. This 32-residue peptide, classified into cysteine framework VI/VII, loop class 6/9/3/3, differs considerably from other δ-conotoxins described in cone snail venoms [67], unlike δ-EVIB, a 29-peptide previously identified in the venom of *C. ermineus* through cDNA cloning [91]. The 3D structure of δ-EVIA solved by NMR (Figure 3B) showed that it assembles into the stable inhibitor cysteine-knot (ICK) motif [95], found not only in other fold C conotoxins from the O1 superfamily but also in toxic peptides from other kingdoms [97]. However, because of its unusually long and disordered 2^nd^ loop, δ-EVIA exhibits a 1:1 cis/trans isomerism in the Leu12-Pro13 peptide bond, which most likely affects the way the toxin interacts with its binding site [67]. δ-EVIA is unique because, unlike most δ-conotoxins already described, it is a selective modulator of neuronal Na^+^ channels in vertebrates, a feature shared only by δ-CnIVD from *Conus consors* [98]. It increased the excitability of frog neuromuscular preparations by increasing the duration of nerve action potentials without affecting those directly elicited in the muscle tissue [90]. Furthermore, it delayed the decay of Na^+^ currents recorded from frog myelinated axons and spinal neurons [90]. The selectivity of δ-EVIA for neuronal Na^+^ channels was confirmed by the observation that only the mammalian neuronal isoforms rNav1.2a, rNav1.3, rNav1.6, and mNav1.7 had their fast inactivation inhibited by the toxin, while that of the skeletal muscle rNav1.4 and the cardiac hNav1.5 isoforms remained unaltered [90, 99]. The interaction between δ-EVIA and the Na^+^ channel was further elucidated when the toxin was found to be active in a chimera formed by the replacement of domains I and/or IV of the muscle Na1.4 by those of the neuronal Nav1.7 [99]. In addition, molecular dynamics and docking data showed that the voltage sensor of domain IV and the 5^th^ transmembrane segment (S5) of domain I form the binding site to δ-EVIA in the Na^+^ channel [99].

A set of eleven conotoxins were identified through a combination of nanoNMR spectroscopy, liquid chromatography, and mass spectrometry in a study focused on the intraspecies variability of the milked venom from eight specimens of *C. ermineus* [100]. Although the actual sequences of these peptides, named EIB, EIB[O8], EIC, EIIB, EIIB[O2], EIIB[O2,8], EIIC, EIIIA, EIVA[P5,7,13], EVIIA, and EVIIA[O22] have not been deposited, some of them appear to differ only in the absence or presence of certain PTMs [100]. It is worthy of note that, at least for the specimens of *C. ermineus* evaluated in the aforementioned study, the composition of the venom varied considerably among different specimens, while remaining fairly constant in individual specimens throughout time [100].

Further insights into this astounding intraspecies variability were obtained through a comparison of the transcriptomes of three specimens of *C. ermineus* from different islands in Cabo Verde, which revealed that only about 20% of the inferred mature conotoxins were present in the venoms of all three individuals [88]. Moreover, venom composition and expression levels varied significantly along the venom duct: in terms of diversity, the distal region was found to be richer, while the proximal region exhibited higher expression levels, particularly of conopeptides from the A superfamily [88]. Although many known and unassigned superfamilies were represented in the transcriptomes of the venom ducts of these *C. ermineus* specimens, members from the superfamilies O1, O2, M, and T were present in larger numbers [88]. The sequences of 11 novel conotoxins - four from the A superfamily (E1.1, E1.2, E1.3, and E4.1), two from the O1 superfamily (E6.1 and E6.2), three from the T superfamily (E5.1, E5.2, and E5.3), one from the D superfamily (E20.1), and one from the E superfamily (E22.1) - were deduced based on their precursors [86] (Table 7). 

In addition to the aforementioned conotoxins, five disulfide-poor conopeptides were identified in the venom of *C. ermineus* by the same transcriptomic analysis [88] and had their precursor-derived sequences published in the ConoServer [26]: conantokin-E2, conkunitzin-E3-E5, and con-ikot-ikot-E1 (Table 7). In a previous study, a conantokin named conantokin-E1 was cloned from the genomic DNA of *C. ermineus* along con-P, an identical conantokin from the venom of the closely related species *C. purpurascens* [92]. These 24-residue conopeptides present five gamma carboxylic glutamic acids and a long inter-cysteine loop in their structures. As con-P was found to be an antagonist of N-methyl-D-aspartate (NMDA) receptors [92], it is safe to assume that conantokin-E1 has the same target. 

Finally, larger proteins were also identified in the venom of *C. ermineus*. The milked venom of this species, whose major protein component was found to be a hyaluronidase named Hyal-E, exhibited fibrinogenolytic and gelatinolytic activity [17]. In addition, an MS analysis identified an angiotensin-converting enzyme-1 (ACE-1) and an endothelin-converting enzyme-1 (ECE-1) in the milked venom of this species [101]. Although their roles in the envenomation need further clarification, the fact that these enzymes are present in the injected venom point to them being relevant in some way. 

## Conclusion

In spite of the many different species of venomous animals, found in almost every ecosystem of our planet - from snails and fish to insects and arthropods, not to mention reptiles - they all have in common the fact that the toxins contained in their venoms have immeasurable biotechnological and therapeutic value, by virtue of their pharmacological targets. Cone snail venoms are not an exception to that rule, for they contain a powerful cocktail of bioactive molecules that target mainly ion channels and membrane receptors, which are crucial players of a number of vital physiological processes. 

But perhaps the most striking feature of cone snail venoms is their unmatched uniqueness. Hundreds of cone snail species have been described to date, and every single one of them produces a different venom containing hundreds of different conotoxins, among other no less important molecules. This diversity is the result of the remarkable ability these animals have to adapt their venom composition to different circumstances imposed by the environment, as well as of the variation in the mature protein sequences of conotoxins from different species. 

In this review, we sought to assemble the information available on cone snail species found in the various biogeographic regions that form the Brazilian coast, focusing on the structural and pharmacological features of the toxins already identified in their venoms. Although only four species, out of the 31 described off the Brazilian coast to date, have had their venoms at least partially explored, a large number of conotoxins and other conopeptides were identified and some of them extensively characterized, attesting to the potential contained in these venoms.

In fact, Brazilian biodiversity has already provided Captopril - a potent angiotensin-converting enzyme inhibitor based on a peptide isolated from the venom of the snake *Bothrops jararaca* - as a successful drug to the market. More to the point, an ω-conotoxin from the venom of the Pacific cone snail species *C. magus* is the biological source of the analgesic Prialt^®^, as previously discussed here. Thus, the thorough study of cone snail species found in Brazil holds considerable promise, for their largely untapped venoms could be the source of another biodiversity-derived drug.
